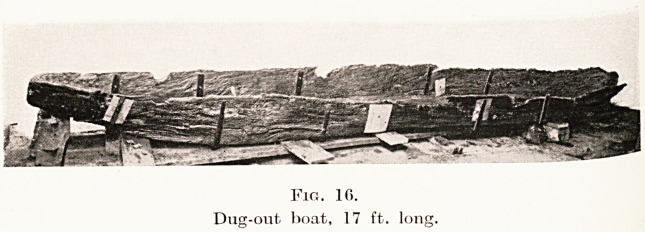# The Twenty-Fifth Long Fox Memorial Lecture: Somerset Lake Villages

**Published:** 1936

**Authors:** A. Bulleid


### The Bristol Medico-Chirurgical Journal

*" Scire est nescire, nisi id me
Scire alius sciret."*

WINTER, 1936.

### THE TWENTY-FIFTH LONG FOX MEMORIAL LECTURE:

DELIVERED IN THE UNIVERSITY OF BRISTOL
ON TUESDAY, OCTOBER 20th, 1936.

THE VICE-CHANCELLOR (Dr. T. LOVEDAY, M.A., LL.D.)
*in the Chair.**

BY

DR. A. BULLEID, F.S.A.,

ON

### SOMERSET LAKE VILLAGES.

---

I AM not aware how many among those present here this evening may have known Dr. Fox intimately, or whether their acquaintance with him may date farther back than the early eighties of last century; but it is with pleasure I look back to the years 1881 to

* In introducing the Lecturer the Vice-Chancellor reminded the audience that the Lecture had been founded in 1904 by his friends

1883, when as a medical student I was permitted to participate in his hospitality on several occasions. Dr. Fox's always kindly and cordial greeting so impressed a shy and nervous youth that it is still remembered with gratitude after the lapse of more than fifty years.

### THE SOMERSET LAKE VILLAGES.

From the earliest ages man has had to consider the question of self-preservation. The invention of primitive weapons, the production of fire, and the provision of some form of habitation were important factors towards this end. His first dwelling-places were caves, rock-shelters and pits, or where these natural formations were not available, booths of branches similar to the mia-mias of the Australian aboriginal. Later, when he became more advanced, he sought the protection of water, and erected dwellings on islands, or on piles near the shores of lakes, river estuaries, and the sea. In Europe pile-dwellings were in use during the Stone and Bronze Ages, and to a less extent the Prehistoric Iron Age. By the time this method of house construction had been introduced to the British Isles iron was in use, and as far as is known at the present time there are no lake-dwellings either in Great Britain or Ireland belonging purely to the stone and bronze periods similar to those discovered in Switzerland. This form of architecture having been adopted in Britain late in the prehistoric times and admirers in memory of Edward Long Fox, M.D. (Oxon.), F.R.C.P., for many years Physician to the Royal Infirmary and Lecturer in Medicine at Bristol University College, in the foundation of which he took a leading part. The lecturer need not be a medical man, but must be resident in Bristol or the neighbourhood, or at least have been a student or a member of the teaching staff of Bristol Medical School, and the subject of the lecture must be "some subject connected with medicine or the allied sciences."

was continued into the historic, and there are records of lake strongholds in Scotland and Ireland being occupied and destroyed even as late as the seventeenth century.

Several references to lake-dwellings were made by writers of the classics. Hippocrates about 400 B.C. mentions such habitations in the locality of the Black Sea. Later Herodotus describes the huts of fishermen built over the water in Lake Prasias, now Lake Takhinos, in Roumelia, where the custom of erecting lake-dwellings appears to have continued uninterrupted to the present day.[1]

The adoption of pile-dwellings seems to have been fairly universal in all parts of the world, except in the continent of Australia. Inhabited pile-dwellings and villages may be seen when visiting Borneo, New Guinea, Central Africa, and Venezuela, *i.e.* "Little Venice," to name a few of the places.

In these countries natives live to-day much in the same way as did the lake-dwellers of Switzerland centuries ago.

The recognition of ancient pile-dwellings in Europe dates back little more than a century, at a time when archæology was not considered seriously, when antiquaries were few, sometimes looked at with suspicion, and often treated as peculiar or eccentric people.

The earliest recorded discovery in Switzerland was about 1829,[2] in Scotland, 1812,[3] and in Ireland public attention was first directed to crannog remains in 1839.[4] No particular notice, however, was taken of these early finds, and it was not until the winter of 1853–54[5] that there was any decided advance or interest shown. The accidental discovery of pile-dwelling remains in Lake Zurich opened a new era in the study of prehistoric archæology, and led to the finding of scores of similar sites throughout the length and breadth of Europe, including the British Isles. With reference to the construction of ancient lacustrine habitations, in the vast majority of cases the foundation or understructure is the only part left for investigation. But the examination of this has produced important results, and has been the means of showing us that many methods were adopted. Among the more notable are the three following:—

1. Houses built on a platform raised on piles above the water.

2. The crannog or artificial island.

3. The construction of rectangular basements of wood, the sides of which were made of beams placed horizontally one over the other, similar to the timbers in a Swiss châlet.

The first method is typically that found in the Swiss and continental lakes. They were generally erected in deep water, and were made by sinking posts vertically into the silt of the lake bed until the upper ends were brought to a level several feet above the surface of the water. On the tops of these posts horizontal timbers were mortised or fixed so as to support a compact and substantial platform. Upon this floor rectangular houses were erected. The upright posts were placed fairly close together, and as some of the villages were several acres in extent the number of piles on one site ran into thousands. It was calculated that at the Robenhausen Settlement at least 100,000 posts were used.

The second kind of lake-dwelling was the crannog, an island either wholly or in part artificial. Crannogs were usually constructed in shallow water or in swamps. The foundation consisted of masses of

### PLATE XIX

(*From photograph by Arthur Bulleid, F.S.A*).

FIG. 1.
Foundation of house built on the log-hut principle.

FIG. 2.
Model of lake dwelling made by Arthur Bulleid, F.S.A.

PLATE XX

Fig. 3.
Hurdle-work and portions of square dwelling, Glastonbury lake village.

Fig. 4.

Ladder, 6 ft. 10¼ in. in length, Glastonbury lake village.

timber, logs, and brushwood, stone and clay, together with heaps of peat, bracken, rush or reed placed on the bed of the lake or swamp surface until a circular island had been raised several feet above the general level of the water. Sometimes a gravel bank or small island was selected as a suitable site, and its area extended artificially to the required size. The diameter of a crannog varied considerably, but was of sufficient area for the erection of one, two, or possibly three dwellings, with enough space around them for the comfort and safety of the inhabitants. The island was usually surrounded with a framework of horizontal mortised beams, and a strong palisade driven into the lake bed. Both of these helped to keep the structure together, the palisading also serving a second purpose as a protective wall. Piles were also driven through the foundation singly or in rows for strengthening purposes. The crannog, although met with on the Continent, is the type of structure found in the British Isles.

The third method of construction on the log-hut principle is of special interest, as a variety of it apparently existed at the Somerset villages. (Figs. 1 and 3.) The occurrence of two rectangular frameworks, and the discarded remains of square habitations can only be explained on the supposition that they belonged to this type of lake-dwelling. Characteristic examples of habitations with this kind of foundation have been met with in Lake Paladru, in France, and in several lakes in North Germany, all of which belonged to the Prehistoric Iron Age.

### GEOLOGY.

It is difficult to describe adequately the life of a prehistoric people and the geographical condition of the country they inhabited without a brief reference to the geology of the district.

The locality under consideration is that part of Somerset situated between the Mendip and Quantock Hills. This area is divided by the smaller ridges, Polden and Wedmore, into three low-lying valleys through which the rivers Axe, Brue and Parret slowly pass. At some remote period these flat tracts of land were estuaries open to the Severn Sea, and submerged by its sand-laden tidal waters at least as far inland as a line drawn from Wells to Glastonbury, Langport and Taunton. (Fig. 8, p. 198.)

Some of this sand was deposited on the floors of the Parret and Brue estuaries, and in course of time formed banks, now known to geologists as the Burtle sand-beds, similar to those seen at the present day at low water in the Bristol Channel. Quite a number of separate Burtle sand-banks have been located in various parts of the turbaries lying north and south of the Polden ridge. The sand is stratified, at some places has the appearance of being wind-blown, and contains myriads of sea-shells. These estuaries being tidal, the sand-banks were frequently exposed. Torrential rains and storms sometimes washed stones and gravel from the adjoining hills, and the swollen rivers sweeping this down to the flats covered the sand with a layer of geological debris. This debris occasionally included bones of animals and fresh-water shells. These having escaped the disintegrating action of the floods were deposited side by side, and subsequently covered by layers of marine-borne sand. Later in geological time the ground gradually rose, and the sand-banks at one time submerged became islands, probably inhabited by neolithic man, as on several of them worked flints have been found. This view is strengthened by the recent discovery of neolithic pottery in the peat near Meare. At the present day some of the smaller sand-banks are occupied by farmhouses and buildings, and several of the larger areas by villages, *i.e.* Chedzoy, Middlezoy, and the Burtles.

The sequence of events, however, was not quite so simple as these notes imply, for parts of this district were subject to intermittent subsidences, and forest lands, together with the evidences of neolithic man, were covered by the sea.

Later still there was another great and important change. When the downward movement of the land ceased, the present coast-line was defined, and the whole physical geography of the locality was transformed. Other factors may have helped the formation of the coast-line, but they were probably of minor importance.

As the coast-barrier developed and the inroads of the sea lessened the salt water in the estuarine hollows on the land side became brackish, and ultimately fresh enough for the growth of water plants and the formation of peat. This, together with mud brought down by the rivers, in course of time filled the depressions. We thus arrive at a period in the history of this low-lying part of the county when the levels of land and water had become stabilized, the Stone Age passed, and the Age of Bronze had given place to the Prehistoric Iron Age.

Let us try to visualize the condition of that part of the Brue valley lying between Glastonbury and the coast as it appeared somewhere about 250 B.C. The Glastonbury Tor and Brent Knoll have always been revered and important landmarks, and when the first lake-dwellings were erected the space intervening between these hills was occupied by several shallow meres. These areas of water, of which Meare Pool was the greater, were surrounded with morasses overgrown with reeds and rushes. This growth, as the ground became less marshy, gave place to thickets of willow and alder, and farther afield as the hills and islands were approached to forest trees, oak, ash, yew, and birch, with undergrowth of maple, hazel, guelder rose and hawthorn. In summer time the meres were bedecked with yellow and white water lilies, and their margins by water crowfoot, yellow flags, and other pond weeds. It was under such conditions and with these surroundings that settlers inhabited the Somerset swamps near Glastonbury and Meare in the Prehistoric Iron Age.

### METHOD OF CONSTRUCTION.

Taken as a whole, the British lake-dwellings are small, artificial islands or crannogs accommodating one or two and at the most three houses. Out of the 300 and more recorded sites none approach in extent the Glastonbury and Meare villages. It is this community grouping of a number of houses that makes the Somerset settlements of special interest, for although they are lake villages and analogous to the Swiss, they differ from them structurally. It appears that the Glastonbury and Meare villages started with a few isolated dwellings, probably built in the traditional manner of crannogs. In course of time the boundaries of these islands were extended and additional houses erected, until ultimately they joined to form collections of from fifty to ninety huts. (Fig. 5.)

There is no reliable guide by which we can tell how many of these dwelling sites were occupied at one and

PLATE XXI

FIG. 5.

Plan of the Glastonbury lake village, showing position of causeway, dwelling-mounds and palisading.

### PLATE XXII

FIG. 6.

Circular stone hearth. (Glastonbury.)

FIG. 7.
Series of eleven superimposed clay hearths and stratified fire ash at left side. (Meare.)

the same time, and to try to estimate the population would be an equally speculative matter.

The Glastonbury dwellings cover about $2\frac{1}{2}$ acres, and those at Meare about double this area. As seen to-day they are represented by low circular grass mounds in pasture fields, the elevation of some being so slight that in passing they may not be recognized by the uninitiated.

With regard to the general construction of the Somerset villages, the first step was the making of a substantial under-structure or foundation. This consisted of layers of timber, logs, or brushwood placed horizontally on the surface of the swamp. This raft was then weighted down by blocks of stone, rubble and layers of clay until a sufficient height had been attained above the water-level. The actual dwelling floor was made of a beaten-down layer of clay. The floor surface was sometimes baked hard purposely, at other times covered with boards of split timber, but usually just the bare, unbaked clay was used.

The huts were made by driving a circular row of upright posts into the clay floor from twelve inches to fifteen inches apart, and afterwards filling the spaces with hurdlework and clay daub. The walls were about six feet high, and the diameter of the huts varied from eighteen feet to twenty-seven feet. The roof rafters were supported at the lower end by the wall and at the upper by a central post, and covered with a thatching of reeds, rushes, or possibly heather. (Fig. 2.) The position of the entrance is shown by a break in the line of wall posts, and sometimes by a timber threshold and doorstep of stone slabs. Near the centre, the highest part of the floor and adjoining the central post, there was generally a hearth of baked clay raised a few inches above the floor level. The hearths were usually circular, with moulded margin, and occasionally paved with stone. (Fig. 6.) On account of the spongy condition of from eight feet to sixteen feet of peat underlying the dwellings, and also from the decay and compression of the under-structure, the huts and floors had an unpleasant habit of gradually sinking. It can be realized that this in itself was the cause of much inconvenience, but when the subsidence took place more on one side of the dwelling than the other the discomfort was doubled. After a time, when the floor approached the water-level of the surrounding swamp, it was necessary to add a new hearth, sometimes a fresh floor, and not infrequently an entirely new hut. This was repeated from time to time, so that in the course of years a mound was gradually built up of superimposed floors and hearths. As many as ten floors lying one over the other have been found in one dwelling-site, and in another the number of superimposed hearths was thirteen. (Fig. 7.) The greatest thickness of clay floors in one mound was nine feet at Glastonbury and six feet at Meare. Every scrap of material used in making the foundation had to be brought to the site from either the adjoining hills at Glastonbury or the raised grounds and Poldens in the case of Meare.

Many of the floors, especially at Meare, have shown no signs of a dwelling apart from the hearth and accumulations of fire-ash on and around it. The huts erected over these, we surmise, were of a less substantial character, and possibly of conical shape like a wigwam.

Some time before the final abandonment of the Meare village the swampy conditions had so changed in the immediate surroundings of the site that clay floors were placed on the hard surface of the peat without the support of either a timber or brushwood under-structure. This fact is of importance when considering the length of the occupation, for the progress of such a change in a swamp is generally slow.

Although the two villages were built and inhabited at the same time there are several structural differences. For instance, the Glastonbury village was surrounded with a palisade, the Meare habitations were apparently without this protection. At Glastonbury there was a causeway leading to a landing-stage; so far nothing of the kind has been met with at Meare. It is, however, the discovery of discarded or lost objects in and around the dwellings that affords the best guide to the date of occupation, and the life and cultural attainments of the inhabitants. We surmise there must have been considerable organization and control in villages of this size, and also a recognized system and division of labour. We have learnt that some of the houses were occupied by people carrying on a particular trade or work; for instance, two dwellings are known to have been inhabited by metal workers, another by a maker of bone and antler implements, a third by a maker of pins and needles, and a fourth was either the abode of a miller or of a dealer in mill-stones.

According to Ptolemy, the territory of the Belgæ extended from Bath and Ilchester in Somerset to Winchester in Hampshire, and included these towns within its area.[6] Although some eighteenth-century maps of Somerset have "The Belgæ" printed in the neighbourhood of Meare Pool, the earlier maps of the county are without this information. It must, therefore, be assumed that there was no traditional reason for its introduction at this spot, but that the later innovation was only due to the swampy ground in the vicinity of the Pool causing a blank space in the map, and affording a suitable position for the words "The Belgæ."

As there is no authority as far as we know for placing the Belgic territory so far west, we are led to assume that the two very decided British earthworks

FIG. 8.

Neighbourhood of Glastonbury, showing positions of lake villages and earthworks.

(Fig. 8), one at Ponters Ball bridging the Glastonbury peninsula, the other crossing the Polden Hills near Butleigh Wootton (both of which run nearly in line north and south with protecting ditches towards the east), were constructed with the intention of limiting the Belgic advance by people inhabiting the swamps and raised grounds farther west. We know that some notable objects of Prehistoric Iron Age workmanship have been found in days gone by on the Polden ridge apart from those discovered more recently at the lake villages, and we are therefore led to believe the above-mentioned earthworks were made by the people inhabiting these lake villages and their relations farming the raised grounds and hills.

We will now consider some lake village discoveries and points of interest regarding the inhabitants.

### HUMAN REMAINS.

The total recorded finds of human remains at Glastonbury are forty, and at Meare, so far as the exploration has gone, twenty. These include five infants buried by inhumation under dwelling floors, five complete skulls and ten incomplete. If these crania represent fifteen of the inhabitants of the villages it may be asked what happened to the limbs and bodies of these people, for they were not discovered. Two of the skulls, after decapitation, had evidently been turned upside-down, and when in this position one had a double-edged spear thrust through the foramen magnum, at the same time notching and fracturing the first vertebra. In the second skull the weapon had been forced through the base in front of the foramen. All the complete skulls show cuts and signs of having undergone rough treatment, and one has an old bone injury which had healed. (Fig. 9.) Among the other human remains discovered are a femur which has been purposely perforated, parts of two cremated bodies, and a circular disc cut from an occipital bone with a central perforation. This object is much worn and polished from use, and was probably an amulet. Without going into details and measurements, it may be mentioned that the skulls are "singularly free from variation, and that they all belong to the oval-headed (mesaticephalic) section of the inhabitants of Britain."[7] They are undoubtedly of good type, for when the late Sir W. Boyd Dawkins happened to be examining one of them he remarked to me: "Excellent! Might have been an Abbot or a Bishop."

### BURIAL.

Judging from the Prehistoric Iron Age cemeteries found at Aylesford in Kent, and at other places in the eastern counties of England, we should expect to find the same kind of cremation burials in the locality of Meare and Glastonbury lake villages. The graves referred to were made with care, and evidently with much respect for the departed, as well as for the carrying out of the customary routine and ceremony then in vogue. As no cemetery has been discovered up to now in connection with either of the villages, nothing can be said positively regarding the form of burial. We do know, however, that the ashes of a cremated body were found outside the palisading at Glastonbury, but whether this was due to an accident or signifies some ceremonial observance cannot be definitely stated.

With reference to the inhumation burials of infants under the dwelling floors which occur at both villages, this may have some superstitious meaning similar to that in Northern India and other parts of the world. "When a child dies it is usually buried under the threshold of the house, in the belief that as the parents tread daily over the grave its soul will be reborn in the family. Children are buried, not cremated. Their souls do not pass into the ether with the smoke of the pyre, but remain on earth to be reincarnated in the household."[8]

### THE CAUSE OF THE ENDING OF THE VILLAGES.

When writing about the human remains found at Glastonbury village the late Prof. Sir W. Boyd Dawkins stated "that the lake village was stormed, and that the inhabitants were massacred, some being decapitated and the heads carried on spears before they were thrown into the morass outside the palisades," and concludes "that the village had been sacked and the population either killed or driven away at some period shortly before the Roman conquest of Britain."[9]

At Meare human remains are being found under very similar conditions, but we also find that the site was occupied well down into the Roman period. At the time the above opinion was given the Professor was labouring under a mistaken impression that all the human remains belonged to one date, and were discovered scattered about on the latest surface-level of the village, whereas in only one or two instances was this the case. There is also no evidence of a general burning of the dwellings in either village. Bearing on this point, we consider it very unlikely that the twenty-two human finds discovered in the timber substructure at Glastonbury could have belonged to inhabitants at a final sacking of the village, because many of them were covered by clay floors which had certainly never been disturbed. Again, a tibia, the ends of which had been gnawed by a dog, found on the second floor of a hut, and a portion of a young adult cranium on the fourth floor of a dwelling could not belong to this date. The same argument applies to the remains found in the morass outside the palisading; for instance, a cranium discovered two-and-a-half feet below the surface of the peat and an occipital bone five feet down could hardly have been parts of individuals who were killed during such an unfortunate ending of the village as the Professor suggests.

That gruesome happenings did take place from time to time is likely if we may judge from the number, distribution, and condition of some of the human bones found. It is also clear that dogs were able to get at human bodies and bring the bones into the dwellings to eat.

However, the cause of the ending of the villages will, in our opinion, be found in geographical changes in the level of the country, or of the adjacent sea coast, making the sites unsafe, rather than a tragic extermination by the hand of man. It is interesting to find that other sites were discontinued for similar reasons; for instance, the settlement of Schussenried had no signs of destruction by fire or sword, and it is supposed that owing to the growth of peat it was voluntarily abandoned by the inhabitants. The ending described by the late Sir W. Boyd Dawkins, although more dramatic, is not substantiated by the latest evidence obtained during the explorations at Meare.

### WHO WERE THE INHABITANTS?

With the present incomplete state of our knowledge it is impossible to say definitely who the people were, or how, or where they acquired the artistic skill with which they were undoubtedly endowed. The whole question of the Celts, their origin, migrations, and expansion is such a difficult and controversial subject that the more we consider it the less inclined we are to venture an opinion. It is known that before the coming of Cæsar to Britain the peoples on the two sides of the Channel were friendly, and that considerable intercourse and exchange was taking place between them, also that the British assisted the Veneti against the Roman aggression. The opinion of the late Sir W. Boyd Dawkins was that the skulls found at Glastonbury belonged to the inhabitants, and that they were Iberians; but the obvious questions arise, were the skulls actually those of village folk, or were they obtained by them from elsewhere? The late M. Henri Hubert[10] thought that after the settlement of the Goidels in the British Isles, whom he associates with the people of the Bronze Age, there were three Celtic colonizations of Britain, namely the Picts, Britons and Belgæ, arriving at intervals in this order. The Picts, the earliest, were pastoral folk, these were followed by the Britons, who were agriculturists, and then much later came the Belgæ. He writes: "Is it to the Belgæ or to the Britons that we must ascribe the building of these curious structures?" *i.e.* the Somerset lake villages. Further, in a footnote he says, "I should ascribe them to the Belgæ." This opinion is not acceptable, and for this reason: If the last Celtic invaders, the Belgæ, occupied territory in Southern Britain as far west as the eastern half of

Somerset, the earthworks that have been mentioned as existing at Ponters Ball, near Glastonbury, and on the Poldens were clearly not made by the Belgæ, but by people who opposed them. (See map on page 198.)

An excavation through the vallum and ditch at Ponters Ball made by the writer some years ago produced the following information. Some fragments of pottery obtained from the old turf line under the vallum were considered by the late Sir Hercules Read to be of Bronze Age date. Pieces of pottery from the lowest part of the ditch were like lake village pottery, but as the fragments did not show any distinctive Iron Age ornament the pottery could not be dated with absolute certainty. But nevertheless it would be nearer the mark to place it under M. Hubert's second period, that of the Britons, rather than to ascribe it to the Belgæ.

With regard to the pottery found at the lake villages, that ornamented with incised patterns, we are able to state that the nearest approach to it in technique and design is to be found in the ceramics of Brittany, and in our opinion it is possible the lake village people were Celtic migrants from that part of Gaul, and a branch of that great Celtic advance from the central parts of Europe.

### WEAPONS.

The inhabitants were apparently adept slingers, and judging from the number of sling stones and clay pellets discovered, the sling must have been in constant use.

At Meare suitable stones were procured from some gravel bed as yet unlocated, but at Glastonbury this ammunition was evidently difficult to obtain, and the baked clay pellet was preferred. (Fig. 11.) Collections of several hundred sling stones are frequently met with either in or near the dwellings, and were always ready and close at hand in case of emergency. The people also possessed iron daggers, swords and spears. A very fine Iron Age bronze sword-sheath was recently found by peat cutters in the locality of Meare. Their armour, consisting of shields and helmets, has been found in other parts of Britain. The only indication of the latter at the lake villages is a little bronze pig or wild boar, which was probably used as a crest surmounting a helmet, and a distinguishing badge of preheraldic days.

### SANITATION.

If the houses had an open space in the roof where the rafters joined the central post ventilation would be satisfactory, but on the other hand smoke may have escaped only through the entrance or under the eaves. There is nothing so far to indicate if the dwellings had windows. The hut entrance was generally wide, and some of the houses must have had doors, because an iron key or latch lifter and also a stone with a pivot-hole were among the objects discovered. It is possible that hurdlework doors afforded protection in some instances.

At Glastonbury discarded and broken objects were thrown over the palisading into the swamp. At Meare no general tilting-place for rubbish has as yet been met with. The surface of the dwelling floors were apparently seldom touched, the accumulations of fire ash from the hearths, bones of animals they ate, as well as glass beads and any other lost objects were all trodden in. This resulted in a compact and sometimes stratified black layer spread over the floor, varying in depth from one to six inches. The systematic removal of household refuse was not undertaken, upon the principle that any little addition helped to raise the level of the dwelling floor: this was important and labour-saving where all the structural materials had to be brought from a distance to the site, and outweighed the evils such accumulations may have created. On the other hand, layers of charcoal and peat ash may have had a beneficial effect as a deodorant, and a primitive attempt at sanitation.

### DRESS.

As no fragment of material has been discovered, we have to be largely guided by what is known of ancient Gaulish costume. The weaving of textile fabrics was undoubtedly carried on at the villages, for parts of at least two primitive looms have been found, as well as the accessories used during weaving. For instance, baked clay loom weights are numerous, also bone bobbins, combs for carding wool, as well as bronze and bone needles for sewing.

Spindle whorls abound everywhere, showing the women were continually occupied in making thread for weaving and sewing. The spindle and whorl were still in use in Scotland and the islands until some sixty years ago, and in Jugoslavia are widely used at the present day.

With regard to the fabrics manufactured at the lake villages, some of them were probably not unlike the Scottish tartan, which is of very ancient origin. Judging from the delicate make of some of the bronze fibulæ[11] discovered, we presume these safety-pins

PLATE XXIII

Fig. 9.
Skulls from Glastonbury lake village.

PLATE XXIV

Fig. 10.
Butcher's block, 3 ft. high.

Fig. 12.
The Glastonbury bronze bowl,
about $4\frac{1}{4}$ in. diameter.

Fig. 11.

Baked clay sling pellet,

$1\frac{1}{2}$ in. long.

were used for fastening correspondingly fine materials of clothing. Toggle-like cloak fasteners made of antler have been dug up as well as bone dress attachments.

*Toilet.*—Near one dwelling pieces of a finely-made wooden box were discovered with its contents. It had been the valued possession of a lady who was, no doubt, particular about her appearance. The box contained a bronze mirror,[12] a pair of tweezers, two wood pins, and some black colouring substance. Not far distant from these was a faceted piece of red hæmatite from which red colouring matter had been rubbed. Tweezers were in use generally, for several have been found at various parts of the excavations. As no combs with the teeth set horizontally have been discovered, like those found on Roman sites, it is more than likely that some of the so-called weaving combs may have been used for dressing the hair. (Fig. 15.) This suggestion is strengthened by the fact that these implements are very numerous, and there are few dwelling-sites that have not produced at least one or two specimens.

*Personal Ornaments.*—From the earliest times there has been a fascination for decorating the body with ornaments. At the lake villages many of the inhabitants were the proud possessors of glass bead necklaces, which included sometimes one of amber or jet. Others preferred a necklet consisting of a double string of small perforated pieces of bone, accurately cut and ornamented with incised circles. The glass beads were of various colours, many of them were of a yellow paste, others blue, green or purple coloured, sometimes ornamented with inset white spirals.[13]

Bracelets of bronze or Kimmeridge shale were worn on the arms and bronze rings on the fingers.

Amulets and charms were also popular, and were probably suspended from the neck or from a safety-pin. These took the form of perforated dogs' teeth, a boar's tusk, or a perforated disc cut from a human skull. Small, circular discs made of tin ornamented with a swastika design were also worn as charms suspended from a brooch or the neck. Solid bronze torques, often highly ornamented, were also worn at this period, but they are rare, and so far the villages have not produced a specimen, although they have been found in or near this locality.

### Games and Pastimes.

A small thin stone slab was found having crossed lines incised on one side, forming a series of thirty squares like a miniature chess board. Its use is not known, but suggests some game, such as noughts and crosses. A wooden draughtsman, made of oak, is among the objects found. On several occasions small, circular, flattened pebbles averaging one inch in diameter, smooth and polished from use, have been met with on the dwelling-floors. They are frequently found singly, but sometimes in groups of twelve, nineteen, or twenty-three. One group was associated with bone dice and a dice-box,[14] also made of bone. These stones were presumably used as counters in some game.

With reference to the dice, as they are brick shaped only four sides are numbered, namely 3, 4, 5 and 6. One die has 6 on two sides. Of course, this may be just an accident, but nevertheless it is a highly-regrettable fact, and may, perhaps, lower the high opinion that has always been held of British morality.

Cock spurs have been discovered on more than one occasion, and the 1935 excavations produced three specimens. From this we deduce that the inhabitants indulged in cock-fighting.

### TRADE AND OCCUPATIONS.

Trade was probably carried on by barter, but there was a British coinage of tin, one of these coins having been found. At a later date Roman coins appear to have been in circulation. Iron bars were also used as currency, they were of recognized different sizes and denominations. A tin weight has been discovered, and from this we assume the people must have had some form of scales. The Glastonbury tin weight in its present condition is 1,962 grains, and allowing for its corroded state, it may have represented half a Roman libra of 5,050 grains. Such weights have been found with late Celtic remains in other parts of England.

*Agriculture.*—Some of the inhabitants, or their relations, were farmers, cultivating the raised grounds and hills in the locality. They owned sheep, goats, cattle, pigs, and horses. Sheep must have been quite numerous, and of two or more breeds. At the Glastonbury site the bones of about 4,000 sheep were dug up. Goats were less common. Next to sheep the bones of cattle (*Bos longifrons*) formed the largest proportion of animal remains. Pig bones were fairly numerous. The horses were slender limbed, of a type best represented by the Exmoor pony, and were undoubtedly brought to the villages for food.

The growing of wheat, barley and beans was carried on extensively, specially wheat and beans. These have been met with frequently on the dwelling floors at both villages. In one hut a dish, upside down, covered a small heap of grain, and a quantity of the cereal was also lying on the floor around it, presumably the contents of an overturned vessel. At another place outside the dwelling area four wheelbarrows full of grain were obtained from a narrow trench, evidently part of the contents of a boat capsized in the swamp.

The cereals, although outwardly perfect, are just small masses of carbon or charcoal—no life remains; they have not been burnt, the condition is probably due to a chemical change brought about by bacterial action.

Another problem has been, why is the oak always ebony-black ? I suppose bog-oak has been dug up in Ireland for centuries, but as far as we know no one has endeavoured to find out the cause of this change of colour. The *Encyclopædia Britannica* does not even mention bog-oak. I would like it known that it is due to the kindness of Dr. O. C. M. Davis, of this University, that we now know the reason for the change is the presence of iron in the peat. Dr. Davis has devoted much time and thought to the examination of wood and peat samples, and I wish to express my indebtedness to him for his valuable and very interesting contribution to the work.

The inhabitants used a wooden push plough for tilling the ground, and spades, the handle-tops of which are exactly similar in shape and size to those in use at the present day. No change has taken place in this respect for 2,000 years. The people also possessed iron reaping hooks and sickles.[15]

### DIET.

A great deal of time must have been devoted to fishing, hunting, and the killing of birds, but mainly for food purposes.

Regarding the diet of the people, the foods consisted of fish, flesh, fowl, milk, cereals, and fruit.

*Fish.*—These included roach, trout, perch, pike, and shad. They were probably caught with nets, many lead net-sinkers having been found. With reference to the nets, it is uncertain if they were made with wool thread or flax. Wool they had in plenty, but flax has not been discovered, although it was one of the earliest cultivated plants. It has been frequently found in Swiss lake-dwelling sites, even those of the Stone Age. No fish-hook has been discovered at the Somerset villages. We know that later, during the monastic period, Meare Pool and the other smaller meres contained quantities of fish, including pike and eels.

*Flesh.*—The domesticated animals, sheep, goat, cattle, and pig, have been mentioned. (Fig. 10.) The wild animals are represented by red deer, roe deer, wild boar, otter, and beaver. The last-named must have been fairly common. The list of wild animals is rather a meagre one, but it must be remembered that the swamps were not a suitable habitat for many, and on the raised grounds and peninsulas in the neighbourhood they were easily trapped, and would be soon exterminated.

*Fowl.*—Some thirty varieties of birds have been met with; naturally the bones of water fowl are most abundant. The list also includes the pelican, bones of both old and young birds, showing they bred in this country at that time. The nearest place where pelicans can now be seen is in the locality of the Danube. Other bird bones discovered are swan, crane, eagle, goshawk, and kite. These were probably killed by slinging.

*Cereals.*—Wheat, barley, and beans. Bread has been found charred in the form of small, flat cakes, somewhat like the modern penny bun. The meal must have contained a considerable quantity of grit and powdered stone. Although the milling stones were generally made of old red sandstone, and other hard gritty stone, one at least was of lias, which is a soft stone and easily ground away. It is probable that grain and beans were sometimes roasted as an aid to milling, and also for storage purposes. Grain has been treated in this way in Germany, Switzerland, Scotland, and the Isles down to a comparatively recent date. Professor Heer thinks that barley found in the Swiss lake dwellings must have undergone this treatment. We learn from other sources that in the time of David[16] "Shobi, Machir, and Barzillai brought parched corn and pulse for David." References are also to be found in the classics regarding the parching of grain.[17]

*Fruit.*—So far the wild fruits form rather a disappointing list, they include the blackberry, dewberry, sloe and hazel nut. What the inhabitants did with the sloe is not known, but in one place the greater part of a wheelbarrow full of sloe stones was obtained immediately outside the palisades.

### MANUFACTURES.

*Pottery.*—Pottery-making was carried on extensively, and was probably undertaken by the women, as was the weaving and spinning. With few exceptions the pots were handmade, the rare examples of wheel-turned vessels were unornamented, and possibly imported. Among primitive peoples in all parts of the world the making of pottery falls to the lot of the women. The same routine appears to be carried out whether it is by the natives in the Congo region of Africa or by the North American Indians, and the method is the same as the lake village inhabitants worked some 2,000 years ago. The operative, after making a flat, circular disc for the base, moulds between her hands long rope-shaped rolls of clay, these are placed near the margin of the base, and then one over the other in spiral form until the vessel is built to the required height and shape. After welding the rolls together the outer and inner surfaces are smoothed and burnished with a bone tool or other implement.

At the lake villages a large proportion of the pots (one in seven) are ornamented with incised, geometric designs. (Fig. 13.) It was customary to ornament the bases as well as the sides of vessels, and the implements used in doing this work have been found. Among these are pieces of pointed antler for drawing lines and making dot-like depressions, stamps for making circles, an antler implement for producing double lines and a cordon at the same time, and a modelling tool of antler of similar shape to a box-wood tool sold at the present day. On some pots certain marks and dots in line were made by a roulette, a small, wheel-shaped implement with a cogged edge. At least three varieties of this were in use. Bone burnishing tools are also among the potter's instruments discovered. Although the pottery was not wheel-turned, some kind of revolving table was apparently used when making girth grooves and lines. Bone implements were also used for moulding the rims and lips of pots. Some of these tools were obtained with other late Celtic remains at Wookey Hole by Mr. H. E. Balch, F.S.A.

*Metal Work and Smelting.*—At least two dwellings were occupied by metal workers. This was shown by the remains of furnaces, pieces of crucibles, fragments of bronze, and bronze dross. A *tuyère* of baked clay, a funnel-shaped object which received the wooden nose of the bellows and conducted the air blast to the furnace, was also dug up. Besides bronze, tin and lead were also smelted. (Fig. 12.) Iron was presumably imported in bar shape from other parts of the country ready for the smith, but as lumps of iron slag have been discovered at the villages it would appear that the people were capable of producing the metal from iron ore. We do not know for certain where the ores came from, but presume the lead was from the Mendips and tin from Cornwall. Iron ore may have been procured from the Brendon Hill mines, or from Priddy and the Nettlebridge Valley on Mendip. The last-named locality is not unlikely, as fireclay was apparently obtained there by the village people, the nearest beds of which are to be found at Nettlebridge associated with the outcrop of coal seams. The tools used in metal work are not numerous, but include several files. One or two stones have been obtained showing casting marks, and numbers of whetstones, some of which have lines and depressions made by sharpening or grinding metal tools.

*Glass.*—The making of glass beads was probably undertaken by some of the inhabitants, but whether

PLATE XXV

FIG. 13.
Somerset lake village pottery.

PLATE XXVI

(*Photograph by Very Rev. Dom. Ethelbert Horne, F.S.A.*)

FIG. 14.
Part of basket or cradle, Meare lake village.

FIG. 15.
Antler weaving comb, 5 in. long.

FIG. 16.

Dug-out boat, 17 ft. long.

the glass was imported in bulk or was actually produced in the villages is not quite certain. Nevertheless a piece of glass slag was discovered, also a crucible with glass adhering to it, which imply working in glass. Some of the beads were inlaid with spirals and waved lines of white paste, and the range of colours through various shades of yellow, green, blue and purple denote not only skill but considerable knowledge in manufacturing chemicals to produce such fascinating results. The beads are of different shapes, some are in the form of rings, others tubular, and a few approach the spherical. The tubular are the more numerous at Meare.

*Carpentry and Turnery.*—The carpentry tools include small iron saws, gouges, adzes, awls, and billhooks; iron nails, rivets, bolts, and wood mallets come under this heading as accessories. Straight-edged chisels of several widths were also used; this fact was ascertained from the cuts remaining on pieces of timber when making mortise holes—for instance the ladder. (Fig. 4.)

Some kind of primitive lathe was worked for turning tubs, cups, bowls, wheel-hubs, and spokes. The outer surface of some of the tubs cut from the solid were highly ornamented with incised and burnt-in designs. Other tubs were stave made, some fitted together with dowels, others with plain edges like a modern barrel and kept in place with bronze hoops.

A considerable amount of time must have been spent in the making of handles for tools, some of which were ornamented with knobs, and also the making of the framework of looms. Boat making was another branch of carpentry. As the boats were cut from the solid tree trunk, this would mean not only much time and labour but a considerable amount of thought and planning. The boat found near the Glastonbury lake village was nearly eighteen feet in length (Fig, 16.), and another more recently discovered on Shapwick Moor, near Meare, twenty-one feet.

*Basket Making.*—Basket making of osiers was another industry carried on at the villages. Portions of several well-made and carefully-designed examples have been met with. (Fig. 14.) On one dwelling-floor, near the hearth, pieces of a long basket were found containing moss; from its shape we presume it may have been part of a cradle.

*Bone and Antler Workers.*—The making of bone needles and pins apparently occupied the time of one inhabitant, as numerous pieces of bone and several broken and unfinished pins were found in one position on a dwelling-floor. Another house belonged to a man skilled in making the handles of knives and other implements, as well as antler tools.

*Milling.*—Milling was another very important occupation. Out of the forty dwelling sites explored at Meare twenty-two had no milling stones. One dwelling produced as many as twenty-four, and two others twenty and seventeen respectively. It would appear from this that there were houses inhabited either by the manufacturers of millstones or that milling was carried out on a large scale for the community. Two kinds of mills were employed, the circular rotary mill or quern and the saddle-shaped. The former, the more advanced type, was more numerous at Glastonbury.

It is hoped that these notes and remarks will give some idea of the life and occupations of the people inhabiting the Somerset lake villages 2,000 years ago. This chapter of prehistoric Britain, as interpreted, is far from complete, many pages have been lost, and time has nearly obliterated the few lines remaining on others. Still, as the exploration of the lake villages proceed we trust additional information may be obtained and some of the gaps made good.

### REFERENCES

[1] *Prehistoric Times,* p. 182, by Lord Avebury, seventh edition. Williams and Norgate, 1913.

[2] *Lake Dwellings of Switzerland,* p. 10, by Ferdinand Keller, translated by John Edward Lee. Longmans, Green & Co., London, 1866.

[3] *Ancient Scottish Lake Dwellings,* p. 17, by Dr. Robert Munro. David Douglas, Edinburgh, 1882.

[4] *Lake Dwellings of Ireland,* p. 23, by W. G. Wood-Martin. Hodges, Figgis & Co., Dublin, 1886.

[5] *Lake Dwellings of Switzerland,* p. 10, by Ferdinand Keller.

[6] *Coins of the Ancient Britons,* p. 39, by Sir John Evans, also *Ancient Britain and the Invasions of Julius Cœsar,* p. 232, by T. Rice Holmes. The Clarendon Press, Oxford, 1907.

[7] *The Glastonbury Lake Village,* vol. ii., p. 681, by Bulleid and Gray. The Glastonbury Antiquarian Society, 1917.

[8] *The Golden Bough,* by Sir James Frazer.

[9] *The Glastonbury Lake Village,* vol. ii., p. 678.

[10] *The Rise of the Celts,* by M. Henri Hubert, p. 212. Kegan Paul, Trench, Trubner & Co., London, 1934.

[11] FIBULÆ—LA TÈNE TYPE: *The Glastonbury Lake Village,* vol. i., pp. 183–203; British Museum, *Early Iron Age Guide,* pp. 51–53, 94–95; Munro's *Lake Dwellings of Europe,* p. 291; Keller's *Lake Dwellings of Switzerland,* 1866, plate lxix.

[12] MIRRORS: *The Glastonbury Lake Village,* vol. i., p. 220: British Museum, *Early Iron Age Guide,* 1925, pp. 121–123.

[13] BEADS: *The Glastonbury Lake Village,* vol. ii., pp. 354–359; British Museum, *Early Iron Age Guide,* p. 120; Wood-Martin, *Lake Dwellings of Ireland,* 1886, p. 122, plate xxvii.

[14] DICE AND DICE BOX: *The Glastonbury Lake Village,* vol. ii., p. 407.

[15] IRON TOOLS: *The Glastonbury Lake Village,* vol. ii., pp. 360–392; Munro's *Lake Dwellings of Europe,* p. 289; Wood-Martin, *Lake Dwellings of Ireland,* p. 67, plate xiii.

[16] 2 Samuel xvii. 27–29.

[17] Virgil, *Georgics* 1, 267.

## Figures and Tables

**Fig. 1. f1:**
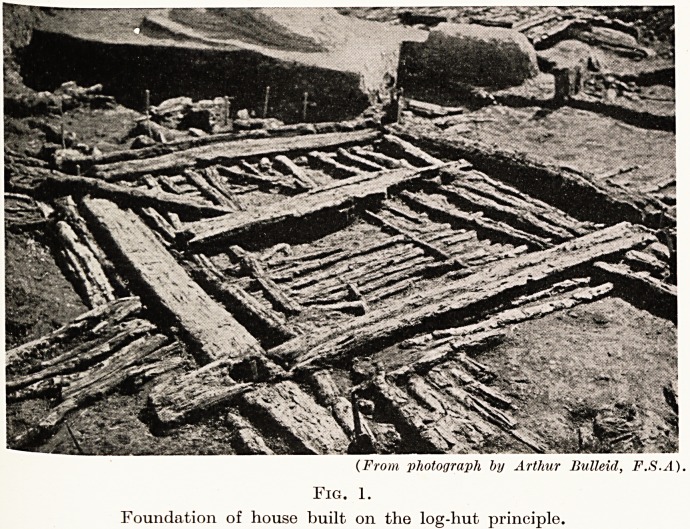


**Fig. 2. f2:**
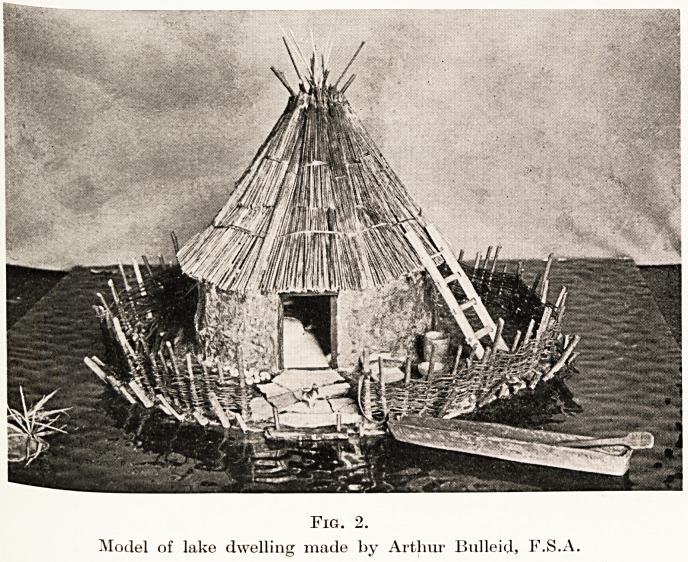


**Fig. 3. f3:**
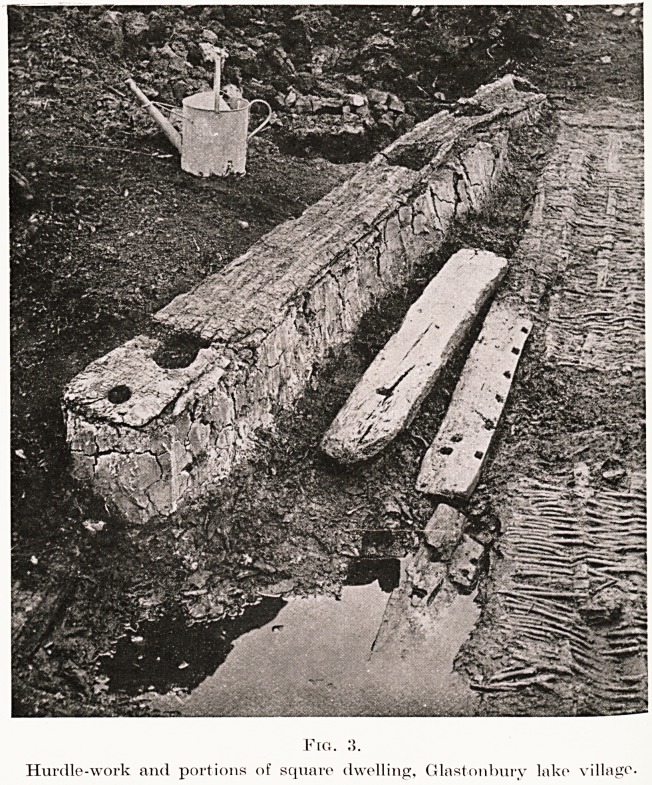


**Fig. 4. f4:**
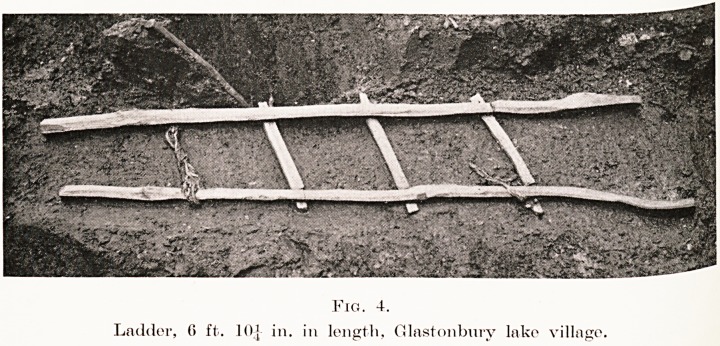


**Fig. 5. f5:**
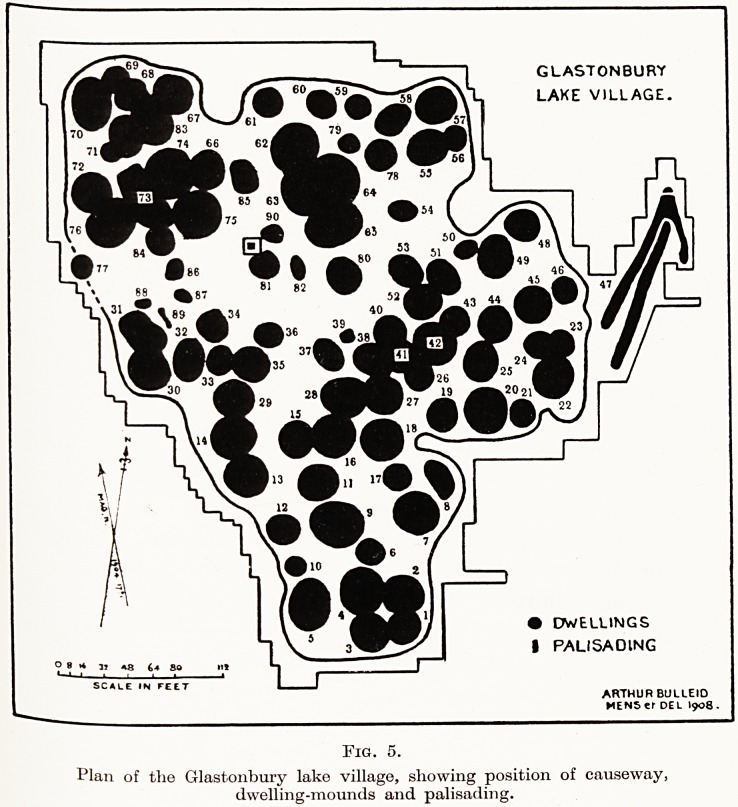


**Fig. 6. f6:**
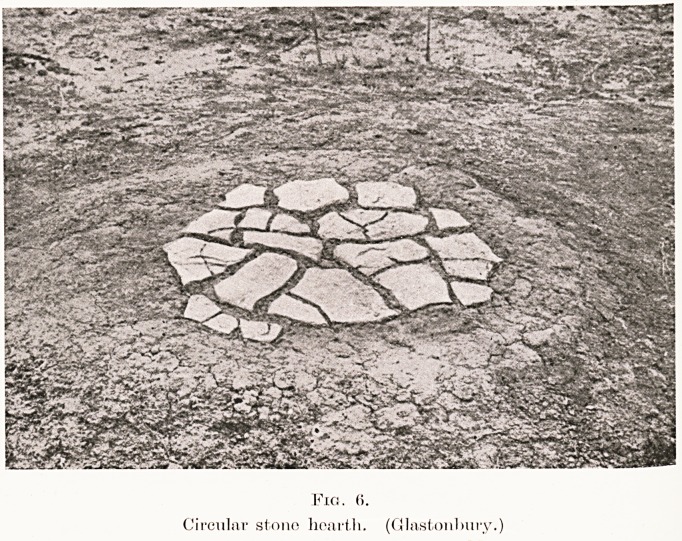


**Fig. 7. f7:**
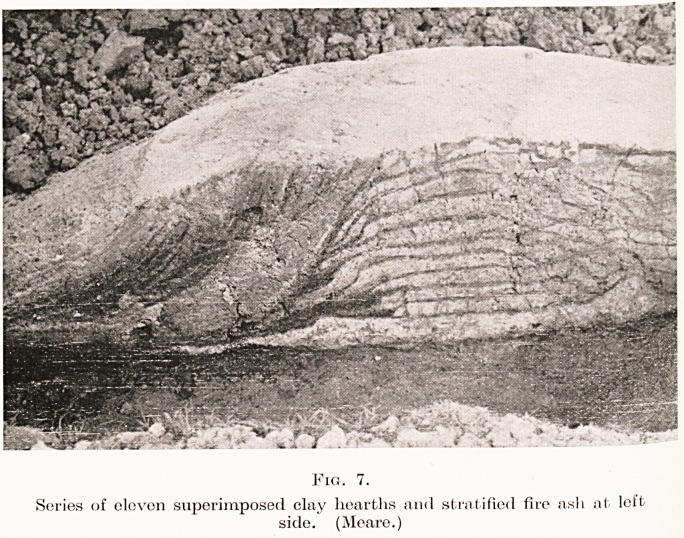


**Fig. 8. f8:**
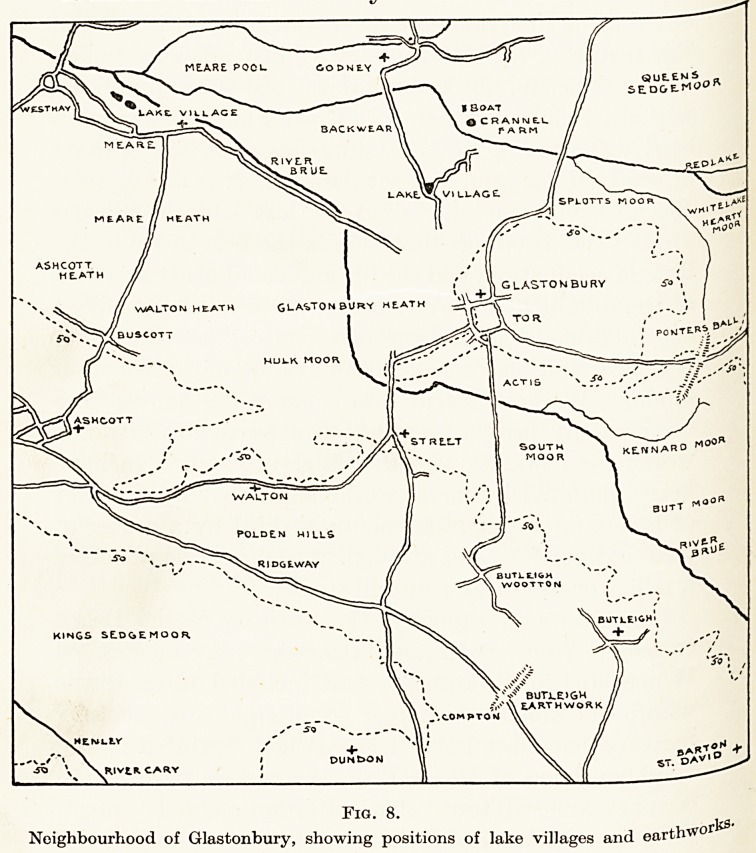


**Fig. 9. f9:**
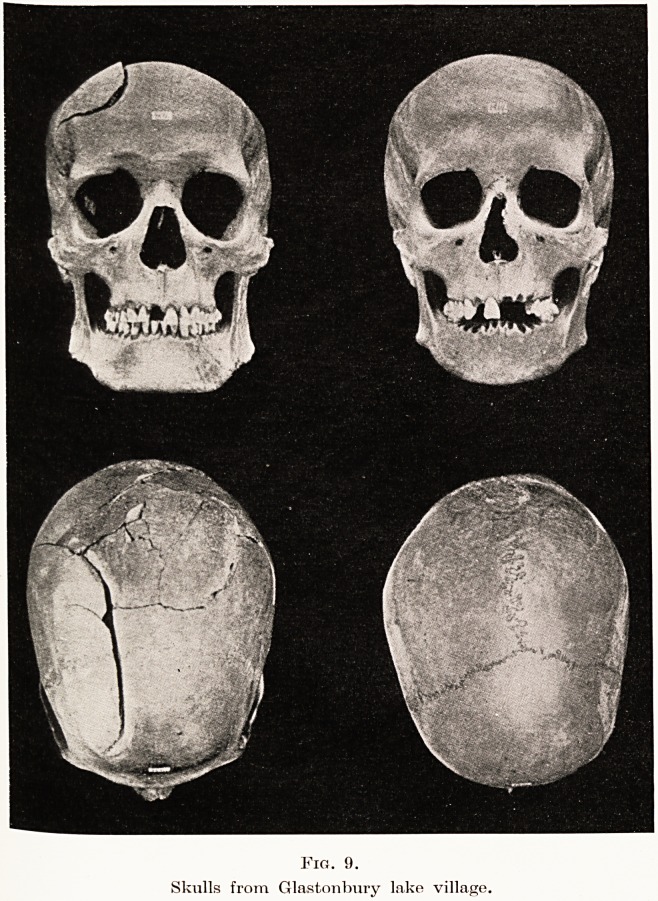


**Fig. 10. f10:**
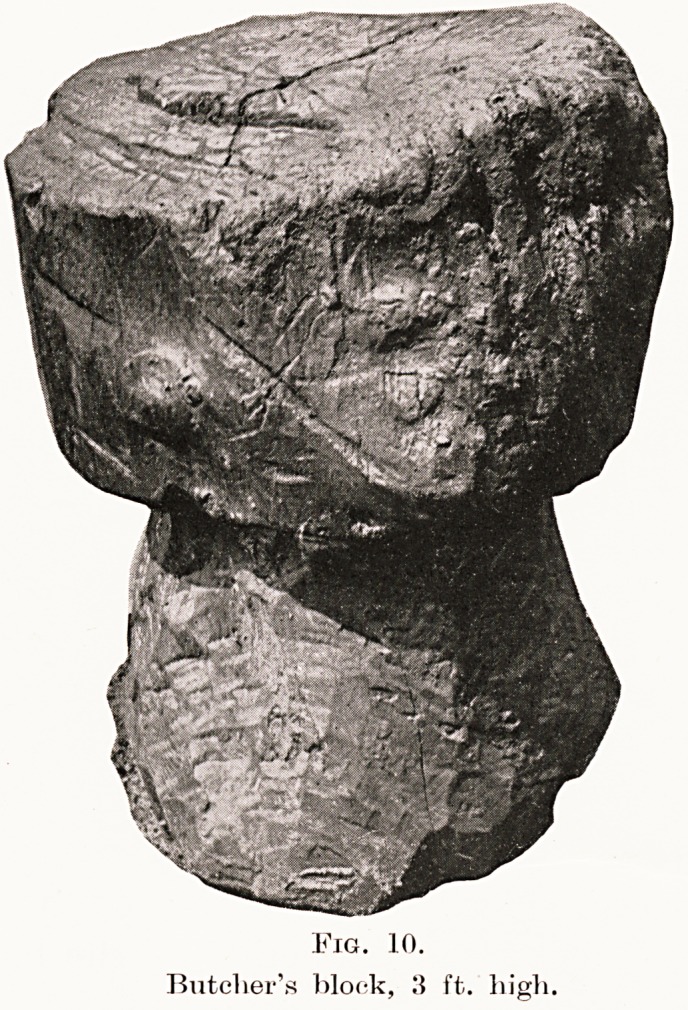


**Fig. 11. Fig. 12. f11:**
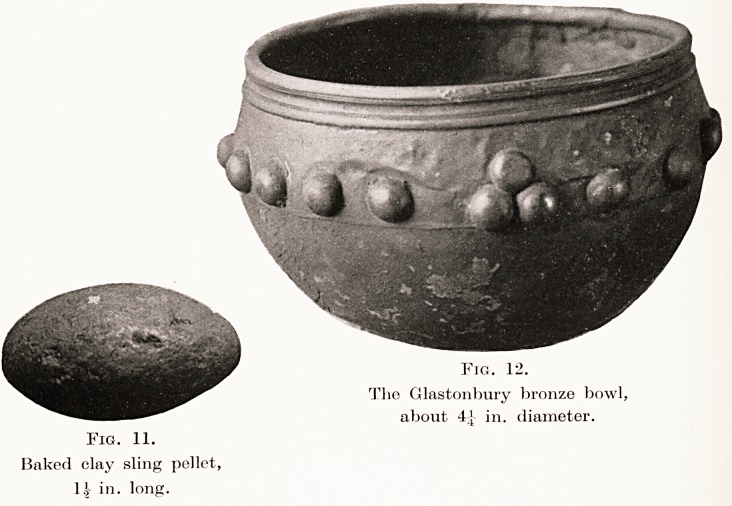


**Fig. 13. f12:**
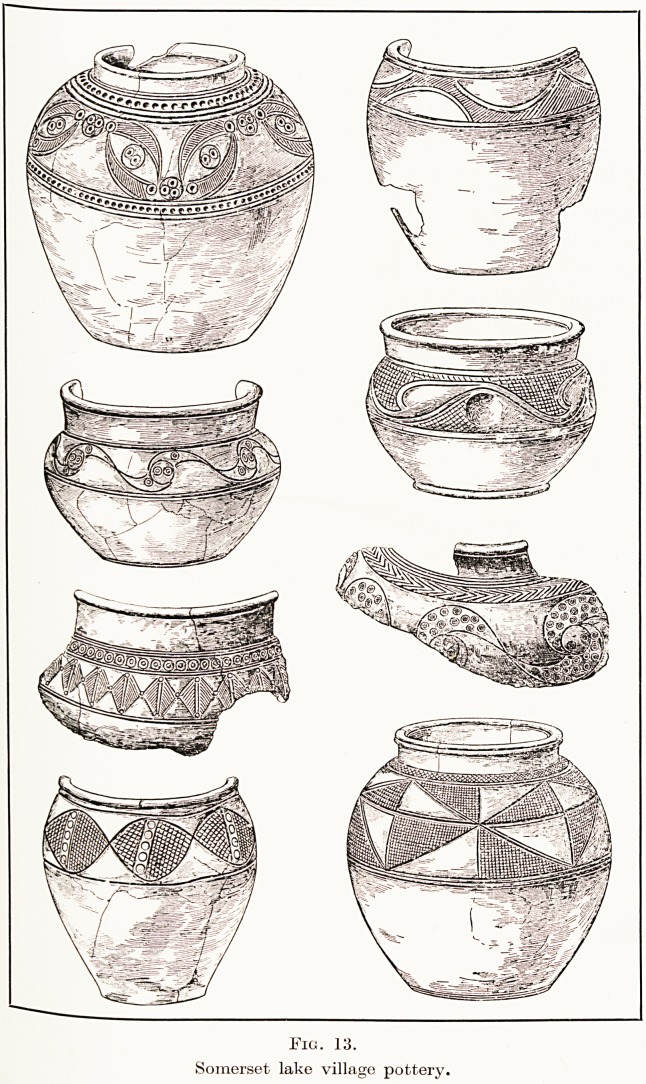


**Fig. 14. f13:**
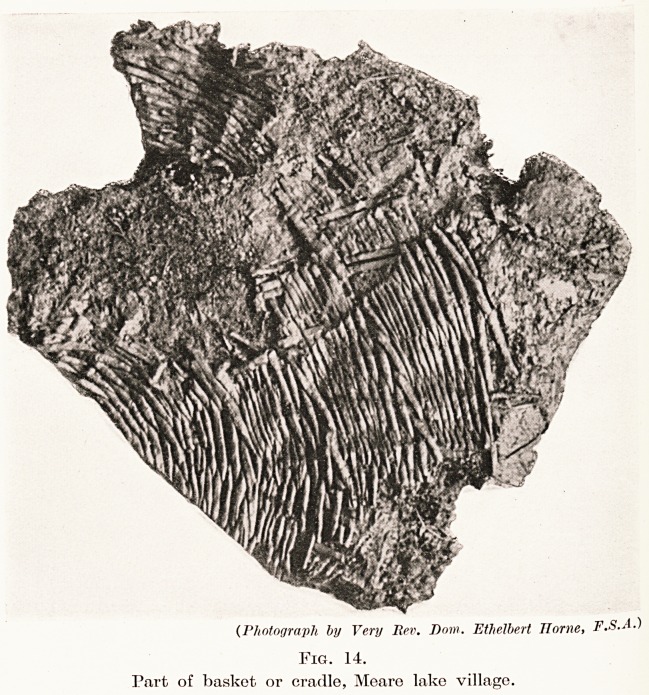


**Fig. 15. f14:**
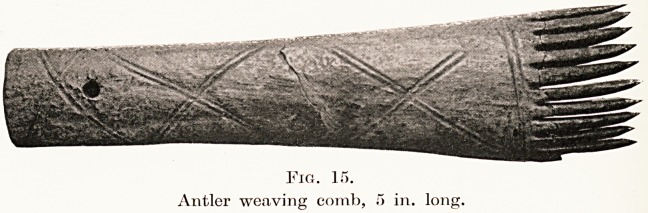


**Fig. 16. f15:**